# Cannabigerol Enhances Muscle Strength and Performance in Dexamethasone-Treated Mice

**DOI:** 10.1021/acsptsci.6c00187

**Published:** 2026-09-30

**Authors:** Izabel Feltrin Fabro, Tatyana Nery, Ana Paula Pinto de Araújo, Amanda Leite Bastos Pereira, Francielly Andressa Felipetti, Aderbal Silva Aguiar Jr

**Affiliations:** † Biology of Exercise Lab (Labioex), Department of Physical Therapy, 28117 UFSC-Federal University of Santa Catarina, Araranguá, Santa Catarina 88905-120, Brazil; ‡ Laboratory of Intrauterine and Fetoplacental Analysis (Laif), Department of Medical Sciences, UFSC - Federal University of Santa Catarina, Araranguá, Santa Catarina 88906-072, Brazil; § Institute for Education and Research (IEP) Remederi, São Paulo, São Paulo 04552-060, Brazil

**Keywords:** cannabinoids, resistance exercise, muscle fatigue, energy metabolism, glucocorticoids, skeletal muscle

## Abstract

Sarcopenia is a major clinical condition associated with aging and is characterized by progressive loss of skeletal muscle mass and function. Resistance training is an effective intervention to counteract muscle atrophy; however, strategies that selectively enhance functional performance remain limited. Cannabigerol, a phytocannabinoid that is not a CB1 receptor agonist and therefore lacks psychotomimetic activity, has emerged as a potential modulator of neuromuscular function, although its role in skeletal muscle adaptation remains poorly understood. Here, we investigated the effects of resistance exercise and cannabigerol, alone and in combination, in a dexamethasone-induced model of muscle atrophy in mice. Animals received dexamethasone (20 mg/kg, i.p.) for 12 days and were subsequently subjected to a 4-week ladder-based resistance exercise protocol. Cannabigerol (10 mg/kg, i.p.) was administered on training days. Resistance exercise restored muscle morphology and improved fatigue resistance. In contrast, cannabigerol acted mainly on neuromuscular performance, increasing muscle power while producing only a small increase in fiber cross-sectional area. Cannabigerol potentiated training-induced power output, increasing performance by ∼55% in the first week and up to ∼87% after 4 weeks compared to exercise alone. In addition, cannabigerol increased peak grip strength (∼10%) and lowered post-exercise blood lactate in sedentary animals (∼48%). These findings show that resistance exercise and cannabigerol exert complementary effects on skeletal muscle—exercise promoting structural recovery and cannabigerol enhancing neuromuscular performance and lowering post-exercise lactate—and support further investigation of cannabigerol as an adjunct to exercise in glucocorticoid-induced muscle atrophy.

## Graphical abstract

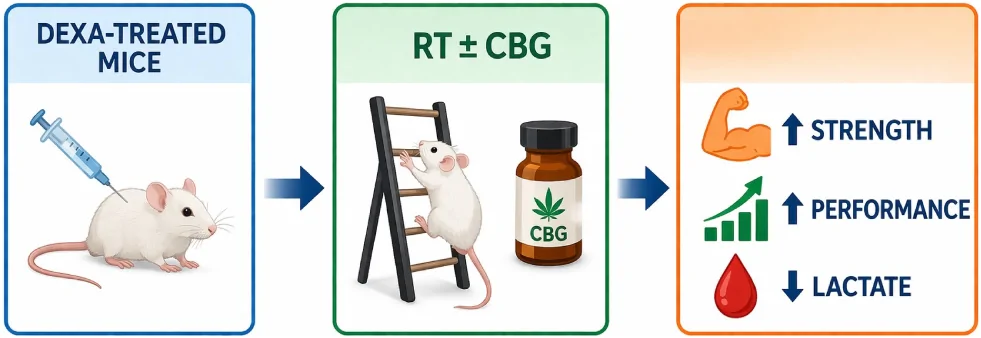

## Introduction

Sarcopenia is a major age-related clinical condition characterized by progressive loss of skeletal muscle mass, strength, and functional capacity.^1,2^ This condition significantly impacts quality of life and increases the risk of disability, morbidity, and mortality. Glucocorticoid-induced muscle atrophy is a well-established experimental model that, although it does not reproduce the aging process underlying age-related sarcopenia, shares core features of muscle wasting—reduced fiber cross-sectional area, impaired neuromuscular function, and metabolic alterations—and is widely used to study muscle atrophy and candidate interventions (see Methods).

Resistance training (RT) is currently the most effective nonpharmacological strategy to counteract muscle atrophy, promoting structural recovery and improvements in muscle performance.^3,4^ However, although RT effectively restores muscle morphology, its capacity to fully optimize neuromuscular efficiency and metabolic responses may be limited, highlighting the need for adjunctive strategies targeting functional performance.

Phytocannabinoids have emerged as potential modulators of neuromuscular and metabolic functions. Cannabidiol (CBD), in particular, has been associated with neuroprotective and neuromodulatory effects, including the regulation of anxiety-related behaviors and neural circuit activity.^5,6^ These findings support a broader role for cannabinoid signaling in central and peripheral mechanisms that may influence exercise performance and fatigue-related processes. In addition, cannabinoid compounds have been implicated in the modulation of inflammatory and metabolic pathways.^7^

Cannabigerol (CBG), a phytocannabinoid that is not a CB1 receptor agonist and therefore does not produce psychotomimetic effects,^8,9^ has received comparatively less attention but displays a broad pharmacological profile, interacting with multiple molecular targets associated with neuronal excitability, inflammation, and energy homeostasis. Despite these properties, its role in skeletal muscle adaptation and exercise-induced functional responses, particularly in the context of resistance training, remains poorly understood. The molecular target profile of CBG is dominated by α2-adrenoceptors, at which it is a highly potent agonist, together with 5-HT_1A_ receptors, PPARα and PPARγ, and TRP channels; CB1 and CB2 receptors are engaged only with low potency and are not preferential targets of CBG.^8–11^

The endocannabinoid system is expressed in skeletal muscle, where the type 1 cannabinoid receptor (CB1) and endocannabinoids regulate energy metabolism, oxidative capacity, and muscle mass.^12^ Genetic and pharmacological manipulation of CB1 modulates muscle phenotype: muscle-specific CB1 deletion increases muscle mass and oxidative fiber content, CB1 antagonism stimulates Akt–mTOR signaling and muscle protein synthesis and attenuates glucocorticoid-induced atrophy, and skeletal-muscle CB1 knockdown reduces grip force and impairs contractile function.^13–15^ Among phytocannabinoids, CBD- and CBG-containing preparations support recovery from exercise-induced muscle damage, and CBG modulates muscle energy homeostasis.^16^ However, most muscle-related cannabinoid research has focused on CB1 signaling in metabolic disease, whereas the role of CBG—which acts largely through PPARα and PPARγ, α2-adrenoceptors, and TRP channels rather than as a classical CB1 agonist—in muscle adaptation and exercise performance remains essentially unexplored. This gap, together with the absence of CB1-mediated psychotomimetic effects of CBG, motivated the present study.

Therefore, the present study investigated the effects of RT and CBG, alone and in combination, in a dexamethasone (DEXA)-induced model of muscle atrophy. We hypothesized that, while RT would promote structural recovery, CBG would selectively enhance neuromuscular performance and metabolic efficiency, thereby improving functional outcomes during exercise. To our knowledge, this is the first study to test isolated cannabigerol—rather than cannabinoid mixtures—as an adjunct to resistance exercise in a model of glucocorticoid-induced muscle atrophy, and the first to dissociate its functional and metabolic effects (muscle power, grip strength, post-exercise lactate) from structural adaptation (fiber cross-sectional area).

## Results and Discussion

### Resistance Training Restores Skeletal Muscle Morphology in DEXA-Treated Mice

Body mass did not differ among experimental groups (F_1,44_ = 0.04; P = 0.83; partial η^2^ = 0.00; Figure 1A). However, sedentary DEXA-treated animals (SED + vehicle) exhibited higher retroperitoneal (visceral) fat mass than naïve animals (t_17_ = 2.59; P < 0.05; Cohen’s d = 1.93; +0.80 g, 95% CI [0.16, 1.44]; Figure 1B). No differences in visceral fat mass were observed among DEXA-treated groups (F_1,44_ = 0.26; P = 0.60; partial η^2^ = 0.006; Figure 1B).

**Figure 1:**
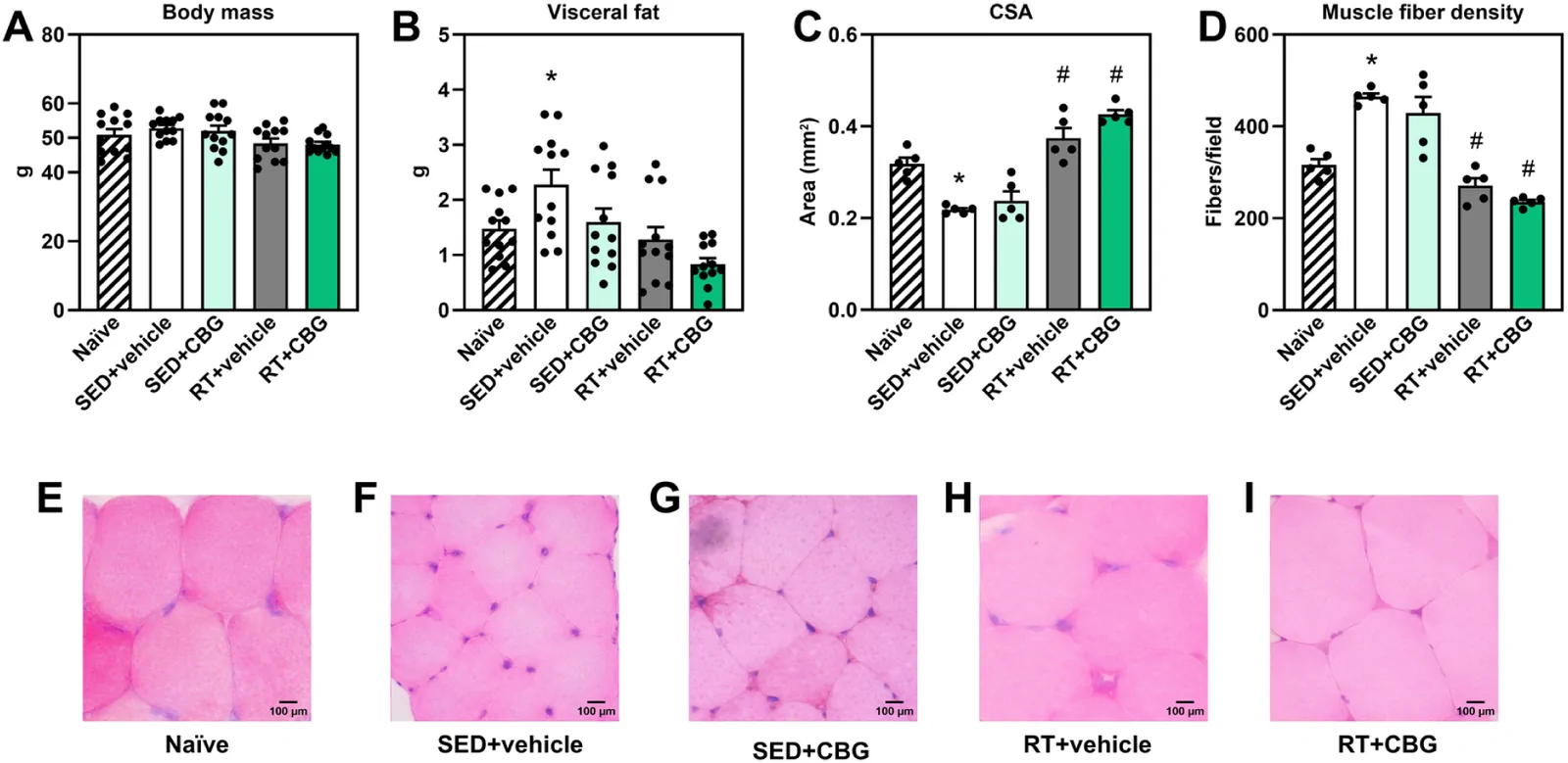
Resistance training restores skeletal muscle morphology in DEXA-treated mice. Body mass did not differ among experimental groups (A). Retroperitoneal (visceral) fat mass was increased in sedentary DEXA-treated animals compared with naïve controls, with no differences among DEXA-treated groups (B). Cross-sectional area (CSA) of quadriceps muscle fibers was reduced in sedentary DEXA-treated animals compared with naïve controls, and resistance training increased CSA in trained groups, with a modest main effect of CBG treatment (C). Muscle fiber density was increased in sedentary DEXA-treated animals and reduced by resistance training, with no significant effect of CBG treatment (D). Representative hematoxylin–eosin-stained sections of quadriceps muscle illustrate morphological differences across experimental groups (E–I). Data are presented as mean ± SEM (n = 8–12 animals per group) from 3–4 independent experiments. *P < 0.05 vs naïve; #P < 0.05 vs SED. Comparisons with the naïve group were performed by Student’s t-test, and comparisons among DEXA-treated groups by two-way ANOVA followed by Tukey’s post hoc test. CBG, cannabigerol; RT, resistance training; SED, sedentary.

Histological analysis revealed significant alterations in skeletal muscle morphology. Cross-sectional area (CSA) of quadriceps muscle fibers was reduced in sedentary DEXA-treated animals compared with the naïve group (t_18_ = 7.11; P < 0.05; Cohen’s d = 4.49; −0.10 mm^2^, 95% CI [−0.132, −0.068]; Figure 1C). In the main experiment, two-way ANOVA revealed a strong main effect of resistance training on CSA (F_1,16_ = 119.53; P < 0.001; partial η^2^ = 0.882). Cannabigerol treatment also produced a significant, more modest main effect (F_1,16_ = 5.24; P = 0.036; partial η^2^ = 0.247; +0.036 mm^2^, 95% CI [0.003, 0.069]). No interaction between resistance training and cannabigerol treatment was observed (F_1,16_ = 1.03; P = 0.324; partial η^2^ = 0.061; Figure 1C).

Muscle fiber density was increased in sedentary DEXA-treated animals compared with the naïve group (t_18_ = 10.22; P < 0.05; Cohen’s d = 6.46; +149 fibers, 95% CI [115, 182]; Figure 1D). In the main experiment, two-way ANOVA revealed a strong main effect of resistance training on muscle fiber density (F_1,16_ = 96.52; P < 0.001; partial η^2^ = 0.858). Cannabigerol treatment did not produce a significant main effect (F_1,16_ = 3.22; P = 0.092; partial η^2^ = 0.167). No interaction between resistance training and cannabigerol treatment was observed (F_1,16_ = 0.00; P = 1.00; partial η^2^ = 0.00; Figure 1D).

### Behavioral, Motor, and Nociceptive Assessment

Open-field analysis revealed no interaction between resistance training and cannabigerol treatment for total distance traveled (F_1,44_ = 0.03; P = 0.86; partial η^2^ = 0.0007; Figure 2A), indicating no differences in locomotor activity among experimental groups. Similarly, no interaction was observed for time spent in the central and peripheral zones (F_1,44_ = 2.22; P = 0.14; partial η^2^ = 0.048; Figure 2B–C), with no significant differences among groups, consistent with the spatial distribution of exploration observed in occupancy plots (Figure 2D).

**Figure 2:**
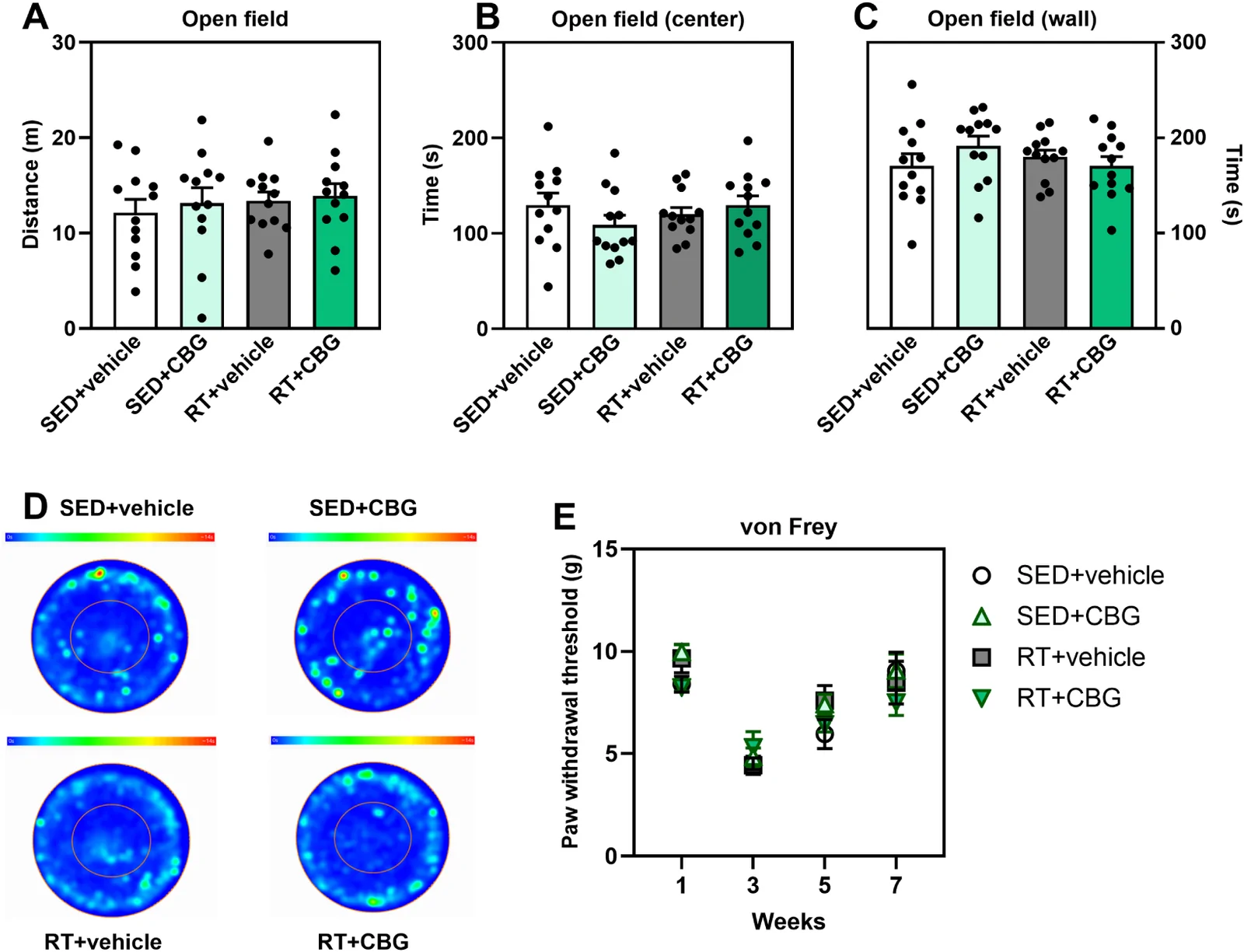
Behavioral, motor, and nociceptive assessment. Total distance traveled in the open field test did not differ among experimental groups, with no significant differences among groups in locomotor activity (A). Time spent in the central and peripheral zones did not differ among groups (B–C). Representative occupancy plots (heat maps) showed a similar spatial distribution of exploration across groups (D). Mechanical nociceptive sensitivity assessed by the electronic von Frey test showed a reduction in withdrawal threshold across the four assessment time points, with no differences among experimental groups (E). Data are presented as mean ± SEM (n = 8–12 animals per group) from 3–4 independent experiments. Two-way ANOVA with repeated measures followed by Tukey’s post hoc test was used. Statistical significance was set at P < 0.05. CBG, cannabigerol; RT, resistance training; SED, sedentary.

Mechanical nociceptive sensitivity, assessed using the electronic von Frey test, showed a significant main effect of repeated measures (Greenhouse–Geisser ε = 0.731; F_2.2,61.4_ = 37.60; P < 0.001; partial η^2^ = 0.573; Figure 2E), reflecting a reduction in withdrawal threshold across the four assessment time points. No interaction between repeated measures and experimental groups was observed (F_2.2,61.4_ = 1.66; P = 0.18; partial η^2^ = 0.056; Figure 2E). As the threshold declined uniformly across all groups, with no differences between them, this reduction most likely reflects the repeated mechanical stimulation during testing rather than a treatment- or model-related effect.

### Neuromuscular Strength Performance

Analysis of peak grip force revealed no main effect of resistance training (F_1,44_ = 0.158; P = 0.69; partial η^2^ = 0.0036; Figure 3A). A significant main effect of treatment was observed (F_1,44_ = 8.21; P = 0.006; partial η^2^ = 0.157; +14.4 g, 95% CI [4.3, 24.6]; ≈10%; Figure 3A), with higher peak grip force in CBG-treated animals. No interaction between resistance training and treatment was detected (F_1,44_ = 0.56; P = 0.46; partial η^2^ = 0.012; Figure 3A).

**Figure 3:**
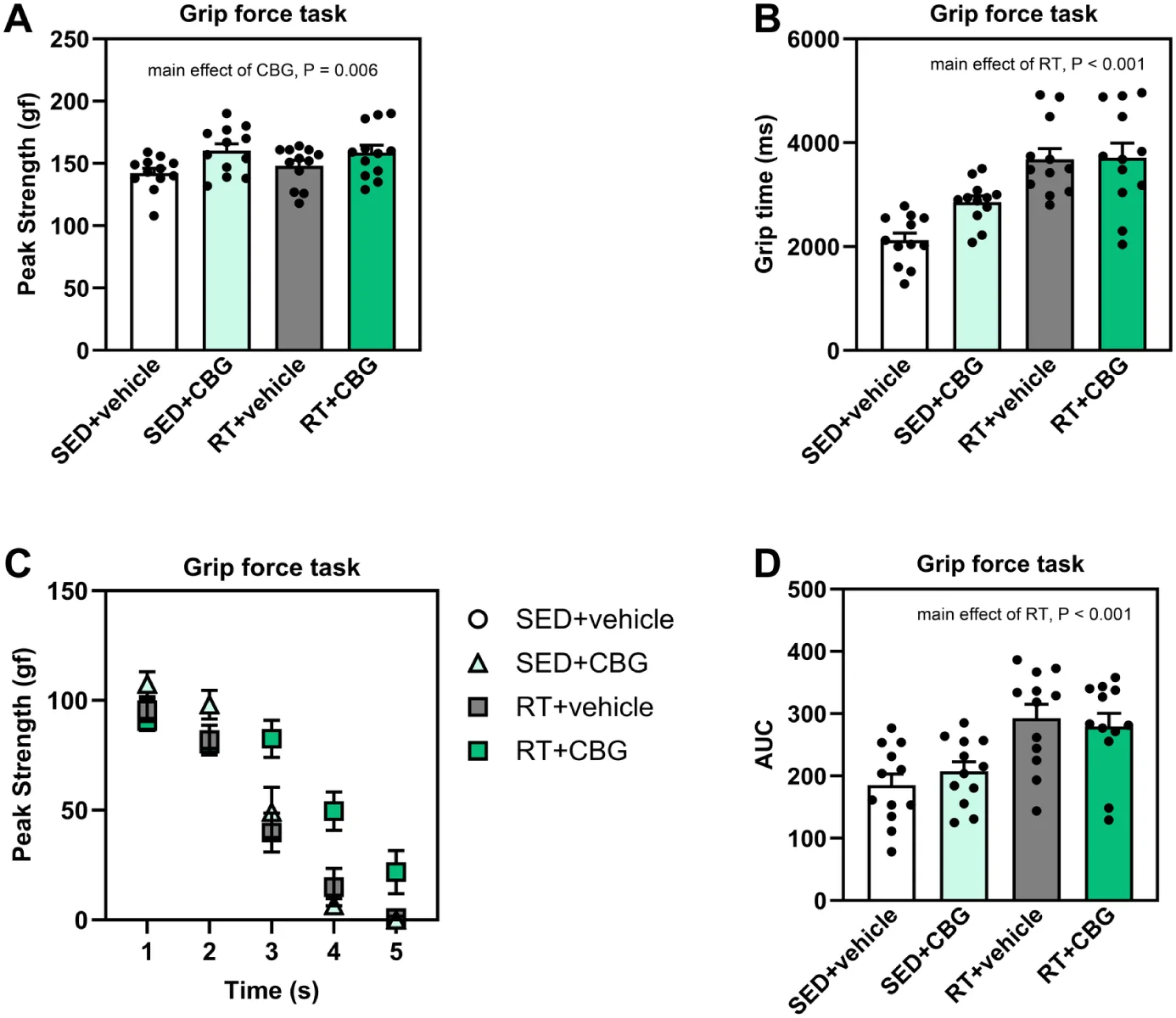
Neuromuscular strength performance assessed by the grip strength test. Peak grip force showed a significant main effect of CBG treatment, with no significant effect of resistance training (A). Grip time was increased by resistance training (main effect of RT), indicating improved grip endurance (B). Grip force declined over the 5-s sustained grip, with trained animals showing greater resistance to fatigue (significant time × resistance-training interaction) (C). Force impulse, quantified as the area under the force–time curve, showed a significant main effect of resistance training, with no significant effect of CBG (D). Data are mean ± SEM (n = 12 per group) from 3–4 independent experiments. Two-way ANOVA (A, B, D) and repeated-measures ANOVA with Greenhouse–Geisser correction (C) were used; significant ANOVA main effects are indicated above each panel. CBG, cannabigerol; RT, resistance training; SED, sedentary.

Grip time showed a strong main effect of resistance training (F_1,44_ = 37.20; P < 0.001; partial η^2^ = 0.458; Figure 3B), indicating improved grip endurance in trained animals. Treatment (F_1,44_ = 3.76; P = 0.059; partial η^2^ = 0.079) and the interaction (F_1,44_ = 3.17; P = 0.082; partial η^2^ = 0.067) were not significant (Figure 3B).

Grip force dynamics over time during the sustained grip revealed a significant main effect of resistance training (F_1,44_ = 18.75; P < 0.001; partial η^2^ = 0.299). A significant repeated measures × resistance training interaction was also observed (Greenhouse–Geisser ε = 0.708; F_2.8,124.6_ = 12.23; P < 0.001; partial η^2^ = 0.217), indicating reduced fatigue in trained animals. No effects of treatment or higher-order interactions were detected (Figure 3C).

The area under the force–time curve showed a significant main effect of resistance training (F_1,44_ = 21.27; P < 0.001; partial η^2^ = 0.326; Figure 3D), indicating increased force impulse in trained animals, with no significant effects of treatment or interaction.

### Muscle Power and Metabolic Responses to Resistance Training and CBG Treatment

Muscle power during the 4-week resistance training protocol was significantly affected by CBG treatment (F_1,22_ = 16.67; P < 0.001; partial η^2^ = 0.431), with greater power output in CBG-treated animals (Figure 4A). A main effect of repeated measures was observed (Greenhouse–Geisser ε = 0.597; F_1.8,39.4_ = 70.71; P < 0.001; partial η^2^ = 0.763), indicating a progressive increase in muscle power across training sessions. A significant repeated measures × CBG treatment interaction was also detected (F_1.8,39.4_ = 9.69; P < 0.001; partial η^2^ = 0.306). Simple-effects contrasts showed greater power in RT + CBG than RT + vehicle at both workloads (60%: +0.89 g·s^–1^, 95% CI [0.31, 1.46], P = 0.008; 75%: +1.73 g·s^–1^, 95% CI [0.91, 2.55], P < 0.001), corresponding to ≈55% greater power in week 1 and ≈87% in week 4 relative to RT + vehicle.

**Figure 4:**
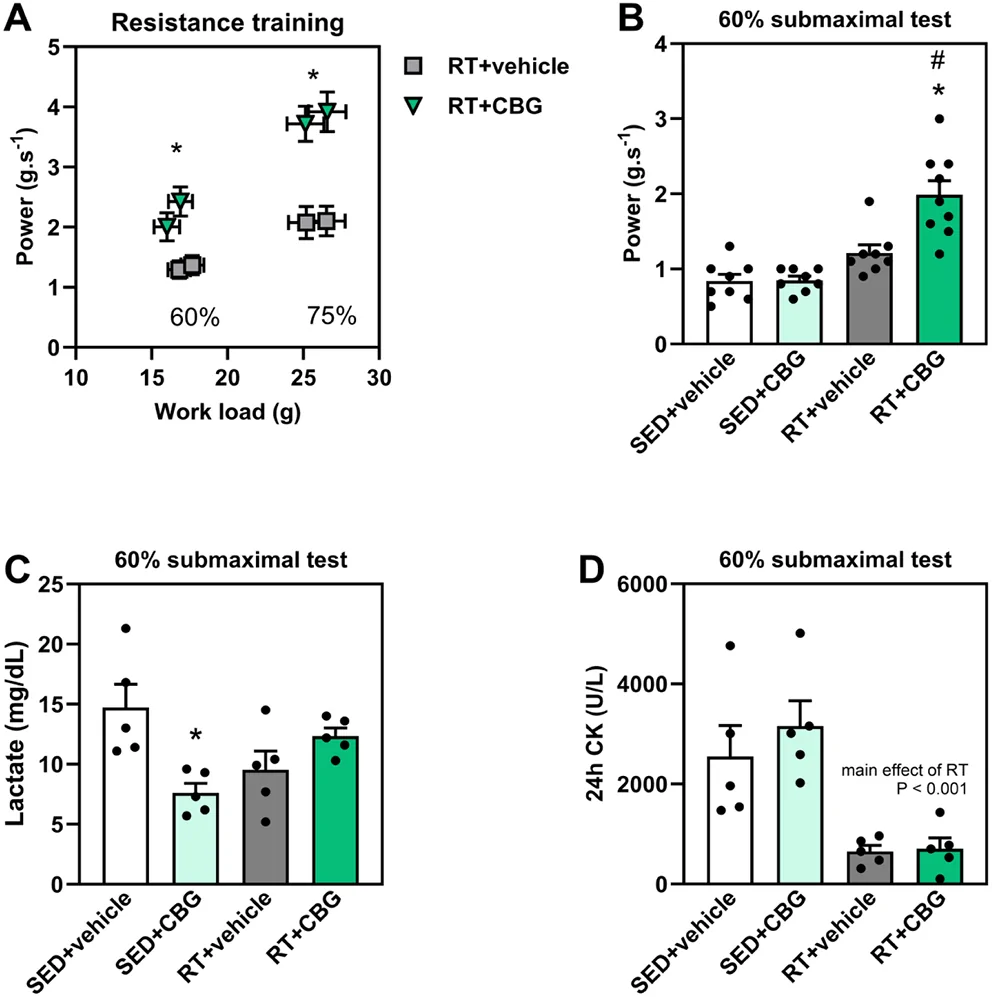
Muscle power and metabolic responses to resistance training and CBG treatment. During training, muscle power was greater in RT + CBG than RT + vehicle at both the 60% and 75% workloads (A; significant repeated measures × CBG interaction). In the submaximal test, RT + CBG produced higher power output than all other groups (B). Blood lactate measured immediately after the submaximal test was lower in SED + CBG than in SED + vehicle (C). Creatine kinase measured 24 h after the submaximal test showed a significant main effect of resistance training, with no significant effect of CBG (D). Data are mean ± SEM (n = 12 per group in A; 8–9 in B; 5 in C and D). Repeated-measures ANOVA with Greenhouse–Geisser correction (A) and two-way ANOVA (B–D) were used; pairwise comparisons used Bonferroni-corrected Welch tests (B) or Tukey’s HSD (C); the significant main effect in D is indicated above the panel. *vs. vehicle; #vs. SED. Statistical significance was set at P < 0.05. CBG, cannabigerol; RT, resistance training; SED, sedentary.

Submaximal performance, assessed at a workload corresponding to 60% of body mass, revealed a significant interaction between resistance training and CBG treatment (F_1,29_ = 9.17; P < 0.05; partial η^2^ = 0.240). Because variance was heterogeneous for this outcome, pairwise comparisons used Bonferroni-corrected Welch tests, which showed that the RT + CBG group exhibited higher power output than all other groups (versus SED + vehicle, P < 0.001; versus SED + CBG, P = 0.001; versus RT + vehicle, P = 0.016; Figure 4B).

Blood lactate, measured immediately after the submaximal test, showed a significant interaction between resistance training and CBG treatment (F_1,16_ = 13.61; P < 0.05; partial η^2^ = 0.460; Figure 4C). Tukey post hoc analysis indicated lower lactate in the SED + CBG group than in the SED + vehicle group (−7.1 mg/dL, 95% CI [−11.9, −2.3]; ≈48% lower; P < 0.05; Figure 4C).

Creatine kinase, assessed 24 h after the submaximal test, revealed a significant main effect of resistance training (F_1,16_ = 27.13; P < 0.001; partial η^2^ = 0.629), with no significant effects of treatment or interaction (Figure 4D).

The present study demonstrates that resistance training (RT) and cannabigerol (CBG) exert distinct and complementary effects on skeletal muscle in a dexamethasone (DEXA)-induced model of muscle atrophy. While RT primarily restored muscle structure, as evidenced by normalization of the fiber cross-sectional area and density, CBG preferentially enhanced functional performance, particularly during exercise.

RT is well-established as a key intervention to counteract muscle atrophy, promoting structural recovery and improvements in muscle function.^3,4^ In line with previous studies, RT effectively reversed DEXA-induced morphological alterations and improved fatigue resistance. However, although RT restored muscle structure, its impact on optimizing neuromuscular efficiency and metabolic performance may be limited, supporting the need for adjunctive strategies targeting functional outcomes.

In contrast to RT, CBG did not substantially alter muscle morphology—producing only a modest increase in fiber cross-sectional area (partial η^2^ = 0.247, versus 0.882 for RT)—yet markedly enhanced functional and metabolic outcomes, increasing muscle power and grip strength and lowering post-exercise blood lactate. This dissociation between strong functional/metabolic effects and a limited structural effect indicates that CBG improves muscle performance primarily through functional and metabolic, rather than hypertrophic, effects.

CBG’s effects were outcome-specific rather than uniformly potentiated by training: its enhancement of muscle power was greatest in combination with resistance training (significant repeated measures × CBG and RT × CBG interactions), whereas its effects on grip strength and fiber cross-sectional area were independent of training (main effects), and the reduction in post-exercise lactate was observed in sedentary animals. The progressive, training-dependent increase in power indicates that CBG facilitates exercise-induced functional adaptation rather than acting as a direct hypertrophic agent.

CBG has been characterized as a partial dual PPARα/γ agonist rather than as a selective PPARγ ligand. In COS-7 reporter assays, CBG activated both mouse PPARα and PPARγ across a 0.1–30 μM range; the PPARα response followed a bell-shaped concentration–response relationship and, at 10 μM, exceeded that of the reference agonist fenofibrate, whereas the PPARγ response was comparable to that of rosiglitazone used as a positive control.^17^ Functional readouts were isoform-divergent: in HepG2 hepatocytes, CBG upregulated PPARα together with its β-oxidation target genes *CPT1A* and *ACOX1*, whereas in 3T3-L1 preadipocytes, it enhanced adipogenesis less efficaciously than rosiglitazone and, unlike the other phytocannabinoids examined, did not raise PPARγ transcript levels.^17^ Subsequent work in bone marrow–derived mesenchymal stem cells confirmed partial agonism at both isoforms within the low-micromolar range and attributed CBG-induced *Ucp1* expression to PPARα engagement.^18^ CBG is therefore best described as a partial dual PPARα/γ agonist, in which the PPARα arm carries the stronger transcriptional output. Neither study examined PPARβ/δ.

The temporal structure of the present study constrains the class of mechanisms that can plausibly account for our findings. CBG was administered on alternate days, and all post-training assessments were performed 48 h after the final dose—approximately 4.8 elimination half-lives (t^1/2^ = 10.0 h)^19^—at which point residual blood concentrations fall below 4% of C^max^. With the exception of muscle power recorded during training and of the von Frey time course, which was assessed longitudinally throughout the protocol, the effects reported here are persistent post-treatment effects rather than consequences of acute target occupancy. Reversible ligand occupancy of α2-adrenoceptors or of TRP channels would not be expected to outlast the compound.^9^ A durable functional phenotype following intermittent, non-accumulating exposure is instead the expected signature of a ligand-activated transcription factor, which is what PPARs are.^20^

The three PPAR isoforms partition the skeletal muscle transcriptional program unequally.^21,22^ PPARγ is expressed in skeletal muscle at markedly lower levels than PPARδ and acts principally on adipogenesis, insulin sensitization, and inflammation.^21^ PPARα drives mitochondrial and peroxisomal fatty acid β-oxidation and, in muscle cells specifically, antioxidant transcription: in C2C12 myotubes and primary human myotubes, commendamide, a *Bacteroides*-derived endocannabinoid-like *N*-acyl amide, activated both PPARα and PPARγ in reporter assays, yet only PPARα activation induced *Gpx4* and *Nrf2*; the effect was abolished by the PPARα antagonist GW6471, was not reproduced by rosiglitazone, and PPARβ/δ was unresponsive.^23^ PPARδ, in turn, is the canonical regulator of oxidative fiber specification and of the adaptive response to training: muscle-restricted PPARδ activation increases type I fiber content, mitochondrial biogenesis, and running endurance,^24^ and the PPARδ agonist GW1516 enhances endurance only when combined with exercise training.^25^

The phenotype observed here—increased muscle power and grip strength, lower post-exercise blood lactate, and only a modest change in fiber cross-sectional area—is metabolic and functional rather than hypertrophic. It maps onto the β-oxidation program that CBG induces through PPARα (*CPT1A*, *ACOX1*),^17^ whose skeletal muscle counterpart (m-CPT1) is under PPARδ control.^21^ The dissociation among our outcomes is itself informative: only the power response was training-dependent, whereas the reduction in post-exercise lactate was present in sedentary animals as well. A training-independent metabolic effect is consistent with direct, ligand-driven transcription of fatty-acid oxidation genes, whereas the training-dependent component of the power response resembles the exercise requirement reported for GW1516.^25^ We emphasize, however, that CBG has never been assayed at PPARβ/δ and that the one endocannabinoid-like ligand tested at this isoform in muscle cells was inactive.^23^ We therefore cannot distinguish between a PPARα-mediated shift toward fatty acid oxidation and a permissive action of CBG on training-induced PPARδ/PGC-1α signaling—a coactivator axis that also suppresses FoxO3-dependent atrogene transcription and thereby opposes glucocorticoid-induced atrophy.^26^

Any PPAR-based interpretation must be reconciled with achieved exposure. After a single 10 mg/kg intraperitoneal dose, CBG attains a peak plasma concentration of approximately 1.24 μM in male CD-1 mice^27^ and a peak whole-blood concentration of approximately 1.63 μM in male C57BL/6 mice.^19^ Equilibrium dialysis places the unbound fraction of CBG in mouse plasma at 0.19, corresponding to a free peak concentration near 0.24 μM.^27^ The large apparent volume of distribution of CBG (>51 L/kg)^19^ therefore reflects extensive protein binding and tissue sequestration rather than enrichment of free drug: the unbound brain-to-plasma partition coefficient is approximately 1.26, indicating near equilibration of unbound CBG across the blood–brain barrier.^27^ The corresponding coefficient for skeletal muscle is unknown, and neither CBG nor its centrally accumulating metabolite cyclo-CBG has been quantified in skeletal muscle. These values cannot be compared directly with the 5–10 μM nominal concentrations at which CBG engages PPARα and PPARγ in vitro,^17,18^ because the free concentration in serum-containing culture medium was not determined and is expected to be considerably lower than nominal for a compound with a calculated logP near 6.4.^27^ A PPAR-dependent mechanism can therefore be neither affirmed nor excluded on pharmacokinetic grounds. Direct measurement of unbound CBG and cyclo-CBG in skeletal muscle is a prerequisite for any definitive mechanistic claim.

Finally, the absence of a substantial hypertrophic response is itself informative and consistent with prior work. CBG was previously assayed on myogenic differentiation of C2C12 myoblasts and found ineffective at concentrations up to 1 μM and inhibitory toward myogenin expression above that range; for this reason, it was not carried into the in vivo arm of that study, in which the phytocannabinoids that improved muscle quality and performance in dystrophic mdx mice were CBD and CBDV, acting through TRPV1.^28^ Our findings agree: CBG did not behave as a myogenic or hypertrophic agent. Improved function in the absence of a commensurate increase in fiber size indicates that CBG facilitates exercise-induced functional adaptation rather than promoting muscle growth. Resolving the responsible isoform will require isoform-selective antagonism (GW6471 for PPARα, GW9662 for PPARγ, GSK0660 for PPARβ/δ), quantification of PPAR target transcripts in muscle (*Cpt1b*, *Pdk4*, *Ucp3*, *Acadl*, *Gpx4*, *Nrf2*), and direct determination of intramuscular CBG concentrations.

Two features of this model bear translation. Corticosteroids remain the standard of care in Duchenne’s muscular dystrophy despite the muscle wasting they produce,^23^ and the dexamethasone model used here reproduces that iatrogenic component. Notably, the phytocannabinoids that improved muscle quality in dystrophic mdx mice were CBD and CBDV, not CBG;^28^ the present data therefore extend the noneuphoric phytocannabinoid literature to a different molecule and to a pharmacologically induced form of atrophy. Whether CBG confers benefit in dystrophic muscle or in patients receiving chronic corticosteroids remains untested.

Although clinical evidence specifically evaluating isolated CBG remains limited, emerging data from human studies using cannabinoid-based formulations provide partial translational support for these findings. A randomized, double-blind, placebo-controlled study demonstrated that a formulation containing CBG improved functional aspects of recovery, including reduced interference of muscle soreness with daily activities following exercise-induced muscle damage, despite minimal effects on objective physiological markers.^29^ Notably, the multicomponent nature of this formulation precludes attributing these effects specifically to CBG, representing an important limitation of the current literature.

Pharmacokinetic studies in male mice show that CBG, at the dose and route used here (10 mg/kg, i.p.), undergoes rapid systemic distribution and clearance, with a low total brain-to-plasma AUC ratio (≈0.26), although the unbound partition coefficient approaches unity (≈1.26),^27^ which is compatible with, but does not establish, a predominantly peripheral action; a central contribution—potentially via the centrally accumulating metabolite cyclo-CBG—cannot be excluded. Because the present study assessed integrated, whole-animal outcomes, it cannot distinguish central from peripheral mechanisms. Likewise, blood lactate was measured at a single post-exercise time point: the lower lactate in CBG-treated sedentary animals indicates reduced post-exercise lactate accumulation—compatible with altered lactate handling or oxidative metabolism, in line with established lactate-shuttle concepts^30,31^ —but does not by itself demonstrate improved metabolic efficiency, which would require time-resolved measurements. Within these limits, the present findings position CBG as a metabolic and neuromuscular modulator that enhances performance-related outcomes independently of hypertrophic adaptation, providing a framework for future mechanistic work.

### Limitations

This study has several limitations. First, it used a glucocorticoid-induced (dexamethasone) model of muscle atrophy, which reproduces several features of sarcopenia but differs from age-related sarcopenia in etiology and time course; the present findings therefore apply primarily to glucocorticoid-induced atrophy, and their extension to age-related sarcopenia requires confirmation in aged models. Second, only male mice were studied, because skeletal muscle hypertrophy and strength responses are more pronounced in males; as sex influences muscle adaptation, nociception, and cannabinoid pharmacology, these findings require confirmation in females. Third, although animals were allocated by simple randomization, outcome assessment was not blinded, introducing a potential for detection bias; this risk is greatest for operator-dependent measures such as histological image analysis and is attenuated for the instrument-based functional and biochemical outcomes (automated grip and power recordings, enzymatic assays). Fourth, the study assessed integrated, whole-animal outcomes without direct mechanistic measurements and therefore cannot identify the molecular target responsible for CBG’s effects (e.g., which PPAR isoform is engaged) or distinguish central from peripheral mechanisms. Fifth, blood lactate was measured at a single post-exercise time point, and the histological analyses were based on small subsamples (n = 5 per group), limiting statistical power for these outcomes. Histological analysis was restricted to the right quadriceps, a mixed but predominantly fast muscle, and fiber typing was not performed; redox-related gene expression in skeletal muscle is not uniform across muscles, with distinct patterns reported for gastrocnemius, quadriceps, and soleus and greater vulnerability of fast glycolytic fibers.^23^ Neither CBG nor its metabolite cyclo-CBG was quantified in skeletal muscle, so unbound intramuscular concentrations remain unknown. No frozen muscle was preserved, precluding molecular end points; the mechanistic interpretation advanced above is therefore hypothesis-generating rather than demonstrated. Finally, secondary outcomes were analyzed without formal correction for multiplicity and should be regarded as exploratory. Mechanistic studies (including receptor antagonism and tissue-concentration measurements), inclusion of female and aged animals, blinded outcome assessment, and time-resolved metabolic assessments will be important to define the therapeutic potential of CBG.

## Experimental Section

### Animals and Ethical Approval

Adult male Swiss mice (outbred; 8–10 weeks old, 45 ± 1.5 g [mean ± SD]) were obtained from the Central Animal Facility of the Federal University of Santa Catarina (UFSC). Animals were housed in groups (up to eight per cage) under controlled conditions (12 h light/dark cycle, lights on at 07:00 h; ambient temperature 21 ± 1 °C) with *ad libitum* access to food and water. Cages were enriched with nesting material and polypropylene shelters, and animals were regularly monitored to ensure welfare throughout the experimental period. All animals were healthy and had not been subjected to previous experimental procedures. All behavioral and functional assessments were performed during the light phase, between 09:00 and 17:00 h. Only male mice were studied because the primary outcomes—skeletal muscle hypertrophy and strength—are more pronounced in males, providing greater sensitivity to detect intervention effects; this restricts generalization to females and is acknowledged as a limitation.

All animal experiments were performed following the protocols evaluated and approved by the Institutional Committee for Animal Care and Use of the Federal University of Santa Catarina, Brazil (CEUA/UFSC) (Ethics Approval Number: 6172240423) and conducted in accordance with the Brazilian Directive for the Care and Use of Animals in Teaching and Scientific Research (CONCEA Normative Resolution No. 55, October 5, 2022). Animals undergoing blood collection were deeply anesthetized with ketamine (100 mg/kg) and xylazine (10 mg/kg) overdose prior to terminal blood collection. Animals not subjected to blood collection were euthanized by an overdose of ketamine (100 mg/kg) and xylazine (10 mg/kg) followed by cervical dislocation, in accordance with CONCEA Normative Resolution No. 37 (February 15, 2018).

## Experimental Interventions

### Dexamethasone-Induced Muscle Atrophy Model

Muscle atrophy was induced by dexamethasone (DEXA) administration, a well-established model that reproduces key features of sarcopenia.^32–38^ Mice received DEXA (Cortvet, 20 mg/kg, i.p.) once daily for 12 consecutive days. Control animals received saline (0.9% NaCl, i.p.) during the same period under identical experimental conditions.

### Cannabigerol Administration

Cannabigerol (CBG) was administered at a dose of 10 mg/kg via intraperitoneal injection. The dose was selected based on prior dose–response studies conducted in our laboratory and supported by the literature.^19,27^ CBG isolate (≥98% purity as determined by HPLC, per the supplier’s certificate of analysis; crystalline powder; Mile High Labs, USA) was dissolved in a vehicle consisting of 95% ethanol, Cremophor RH 40, and saline (1:1:18, v/v/v). CBG or vehicle was administered exclusively during the 4-week training period, on alternate days, coinciding with training sessions for the RT groups; sedentary groups received the same schedule without exercise. All post-training assessments—grip strength, open field, the submaximal exercise test, blood lactate, and creatine kinase—began 48 h after the last CBG dose. The von Frey test was instead performed longitudinally, at weeks 1, 3, 5, and 7 of the protocol (Figure 5). Reported elimination half-lives after 10 mg/kg (i.p.) in male mice range from ∼46 min in plasma to 10.0 h in whole blood; based on the more conservative estimate, 48 h corresponds to approximately 4.8 elimination half-lives, at which point residual concentrations are below 4% of Cmax,^19,27^ and no CBG was administered on assessment days. Consequently, with the exception of muscle power recorded during training (Figures 4A and 5), all measurements reflect persistent post-treatment effects rather than the acute presence of CBG.

**Figure 5:**
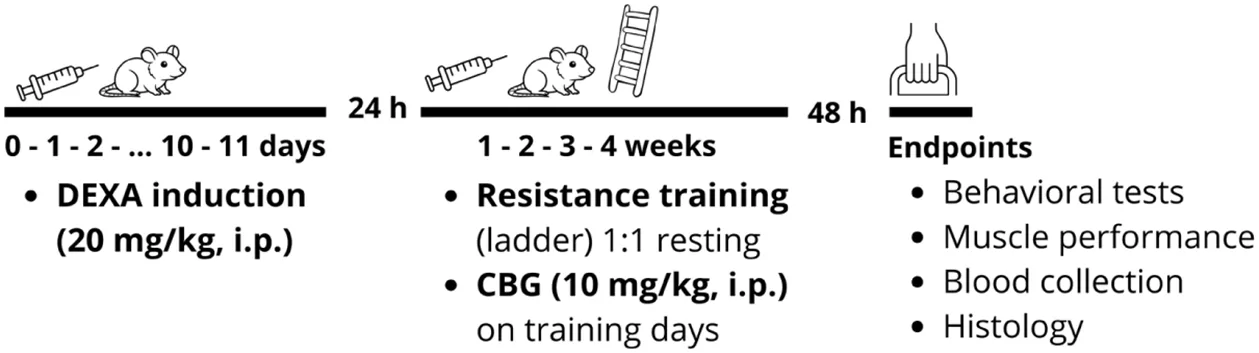
Experimental design. Mice received dexamethasone (DEXA, 20 mg/kg, i.p.) once daily for 12 consecutive days to induce muscle atrophy. After a 24 h interval, animals were subjected to a 4-week intervention period consisting of resistance training (RT) performed on alternate days (1:1 training-to-recovery ratio) and treatment with cannabigerol (CBG, 10 mg/kg, i.p.), administered on training days. Resistance training was conducted using a ladder-climbing protocol. 48 h after the final session, animals underwent end point assessments including behavioral tests, muscle performance evaluation, blood collection, and histological analysis. CBG, cannabigerol; DEXA, dexamethasone.

### Resistance Training Protocol

Following completion of the 12-day DEXA administration period, animals underwent a four-week resistance training (RT) protocol after a 24 h interval. Training sessions were performed on alternate days (48 h between sessions), yielding a 1:1 work-to-recovery ratio. Training was conducted using a vertical ladder apparatus (110 cm height, 15 cm width, 80° inclination, and 5 mm grid spacing; Bonther, Brazil), equipped with a top chamber to allow voluntary entry upon completion of the climb, as previously described in rodent resistance-training models.^39,40^

Animals were first habituated to the ladder for three consecutive days. During this period, mice performed six climbs per day while carrying an empty Falcon tube attached to the proximal portion of their tails.

The maximal carrying capacity was determined using a maximal load test adapted from established ladder-climbing protocols.^39^ Animals performed an initial climb with an empty Falcon tube, followed by a load equivalent to 75% of their body mass. Additional loads of 5 g were incrementally added after each successful climb until failure, defined as the inability to complete the climb after three consecutive attempts. A 60 s rest interval was allowed between climbs. The highest load that was successfully completed was defined as the maximal load.

The training intensity was individualized for each animal based on its maximal carrying capacity. Loads were set at 60% of maximal load during the first 2 weeks and increased to 75% during the final 2 weeks. The maximal load was reassessed at the end of the second week to adjust the training intensity. Each training session consisted of ten ladder climbs with a 60 s rest interval between climbs. Loads were secured to the base of the tail by using an adapted Falcon tube and adhesive tape. Animals were motivated to climb by the presence of a dark shelter at the top of the ladder.

### Experimental Groups

Following DEXA treatment, animals were allocated to experimental groups by simple randomization. All animals in the main experiment received DEXA according to the model described above. The study followed a 2 × 2 factorial design with treatment (vehicle or CBG) and exercise (sedentary [SED] or resistance training [RT]) as factors. The experimental groups were vehicle-SED (n = 12), CBG-SED (n = 12), vehicle-RT (n = 12), and CBG-RT (n = 12). Only DEXA-treated animals were included in the factorial statistical analysis. The experimental unit was defined as an individual animal.

Additional control groups were included for baseline comparison. A sedentary vehicle-treated group that did not receive DEXA served as a physiological control (vehicle-SED control; n = 12). A naïve group was included for histological reference and did not receive experimental interventions (n = 12).

A schematic representation of the experimental design is shown in Figure 5.

### Outcome Measures

The coprimary outcomes of the study were muscle power assessed during the RT protocol and muscle strength evaluated by the grip strength test. Secondary outcomes included behavioral parameters, nociceptive sensitivity, biochemical markers, and histological analysis.

### Behavioral Assessment

Mice were allowed to explore an unaccustomed circular open-field arena (60 cm diameter, 50 cm height; Bonther, Brazil) for 5 min. Locomotor activity was recorded and analyzed using the ANY-maze video tracking system (Stoelting Co., USA), which provided measures of total distance traveled, time spent in the central and peripheral zones, and occupancy plots (heat maps).

Mechanical nociceptive sensitivity was assessed using an electronic von Frey test (EFF301W, Insight, Brazil). Animals were placed on an elevated wire-mesh platform within individual acrylic chambers and allowed to acclimate before testing. Mechanical stimuli were applied to the plantar surface of the hind paw using a calibrated electronic von Frey apparatus. The paw-withdrawal threshold was recorded for each animal.^41,42^

### Functional Performance Tests

Muscle power was assessed throughout the 4-week resistance training protocol during selected training sessions. Power output was calculated for each climb based on the ratio between the total mass lifted (body mass plus attached load) and the time required to complete the climb and was expressed as g·s^–1^. Because each climb spanned the full length of the ladder, the vertical displacement was constant; the mass lifted per unit time is therefore directly proportional to mechanical power output (P = m·g·h·t^–1^) and is used here as its surrogate, reported in g·s^–1^ rather than in watts. Time to complete each climb was recorded, and power was calculated for individual climbs. For each session, power output was averaged across climbs to obtain a representative value per animal. These values were used to assess changes in muscle power across training sessions and to evaluate the effects of resistance training and CBG treatment over time.

Muscle strength was assessed using the forelimb grip strength test, as previously described.^43–45^ Mice were gently suspended by the tail and allowed to grasp a triangular grid connected to a grip strength meter (Bonther, Ribeirão Preto, SP, Brazil; 5 kgf capacity; REF: CHHQBQ0215) using the forelimbs. The animal was then steadily pulled backward along the horizontal axis until release of the grid. Each trial lasted approximately 2–3 s, and peak grip force was recorded using the Grip Strength Meter software interfaced with the apparatus. Multiple consecutive trials were performed for each animal. In addition to peak force, grip time and force–time curves were recorded, allowing the calculation of force impulse (area under the curve). Fatigue was assessed based on the decline in force across repeated trials. The highest peak force value was used for primary analysis, whereas grip time, force impulse, and force decay across trials were used as secondary measures of endurance and fatigue resistance.

A submaximal exercise test was performed after the completion of behavioral and functional assessments. The test was conducted using the same ladder climbing apparatus and procedures employed in the resistance training protocol. Animals carried a load corresponding to 60% of their body mass, attached to the base of the tail by using an adapted Falcon tube. Each mouse performed six consecutive climbs from the base to the top of the ladder with a 60 s rest interval between climbs. This workload was selected to represent a standardized submaximal intensity for the assessment of exercise performance. Immediately after completion of the sixth climb, blood samples were collected for lactate determination. Creatine kinase (CK) levels were measured 24 h after the submaximal test to assess the muscle damage.

Outcome assessments were primarily obtained using automated systems, while manually derived measures were based on standardized procedures that were consistently applied across all experimental groups.

### Biochemical Analysis

Blood samples were collected immediately after the submaximal exercise test by submandibular vein puncture into sodium fluoride/EDTA tubes (glycolysis-inhibitor tubes; BD Vacutainer, ref. 367587). Samples were centrifuged to obtain plasma, which was used for lactate determination. Lactate levels were quantified using a commercial assay kit (MAK064, Sigma-Aldrich, USA) according to the manufacturer’s instructions, and absorbance was measured at 570 nm using a microplate reader (Kasuaki, Japan).

24 h after the submaximal test, blood samples were collected by cardiac puncture under anesthesia for creatine kinase (CK) determination. CK activity was measured by a kinetic method using an automated biochemistry analyzer (LabMax 240 Premium, Labtest Diagnóstica, Lagoa Santa, MG, Brazil) with the Labtest CK-NAC reagent kit.

After euthanasia, the retroperitoneal (visceral) adipose tissue and the right quadriceps muscle were carefully dissected and weighed (g) on a precision balance. The retroperitoneal fat mass was used as an index of visceral adiposity.

### Histological Analysis

The right quadriceps muscle was collected after euthanasia (5 per group) and fixed in 10% buffered paraformaldehyde for 48 h. Samples were processed by using a histological processor (Oma DM-40) and embedded in paraffin by using a tissue embedding system.

Transverse sections (5 μm thick) were obtained using a semi-automatic microtome (Lupetec MRPSA 2016) and stained with hematoxylin and eosin (H&E). Images were acquired using a light microscope.

For morphometric analysis, five non-overlapping fields per section (right, left, upper, lower, and central regions) were analyzed. Twenty muscle fibers were randomly selected in each field, totaling 100 fibers per animal. The muscle fiber cross-sectional area (CSA) and fiber density were quantified.

### Animal Losses and Replacement

During the experimental procedures, a total of five animals were excluded. Two animals were excluded due to a technical error during DEXA administration. Two animals presented severe visual impairment prior to the experimental procedures and were humanely euthanized. One animal exhibited progressive weight loss during the experimental period and was euthanized; a necropsy revealed liver abnormalities. All excluded animals were replaced to maintain the predefined sample size, and therefore, these losses did not affect the final number of animals per group or the statistical analyses.

### Statistical Analysis

Data are presented as mean ± SEM, where n represents the number of animals per experimental group. Sample sizes were based on previous studies using similar models and outcomes.^32–38^ A sensitivity analysis indicated that the main factorial design (n = 12 per group; total N = 48) provided 80% power (α = 0.05) to detect large effects (Cohen’s f = 0.41; partial η^2^ ≈ 0.15) in the principal between-group comparisons, whereas the histological subset (n = 5 per group) was powered to detect only very large effects (partial η^2^ ≈ 0.31); the limited histological sample size is acknowledged as a constraint. Statistical analyses were performed using Statistica version 13.5.0.17 (TIBCO Software Inc., Palo Alto, CA, USA).

Data normality was assessed using the Shapiro–Wilk test; no significant departures from normality were detected, although the sensitivity of this test is limited at the small sample sizes used for histological outcomes (n = 5). Homogeneity of variance was assessed using Levene test (Brown–Forsythe, median-centered). Parametric data were analyzed using Student’s t test, one-way ANOVA, or two-way ANOVA, as appropriate. For repeated measurements, repeated-measures ANOVA was used; sphericity was assessed with Mauchly’s test, and the Greenhouse–Geisser correction was applied to the degrees of freedom when sphericity was violated. Pairwise comparisons used Tukey’s HSD test, except for outcomes with heterogeneous variance, for which Bonferroni-corrected Welch tests were used.

Effect sizes were calculated as Cohen’s d for t tests and partial eta squared (η^2^) for ANOVA models and are reported with 95% confidence intervals for the corresponding mean differences. Two co-primary outcomes—muscle power during training and grip strength—were pre-specified; both remained significant under a Bonferroni-adjusted threshold (α = 0.025). Secondary outcomes were analyzed without formal correction for multiplicity and are therefore considered exploratory.

Statistical significance was set at P < 0.05.

### Safety

Dexamethasone, cannabigerol, ketamine, and xylazine were handled by trained personnel using standard personal protective equipment and in accordance with the institutional procedures. No unexpected, new, or significant hazards or risks were encountered during the course of this work.

## Conclusion

In a dexamethasone-induced model of muscle atrophy, resistance training and cannabigerol exerted distinct and complementary effects on skeletal muscle. Resistance training was the principal driver of structural recovery, normalizing fiber cross-sectional area and density, whereas cannabigerol enhanced functional and metabolic outcomes—muscle power, grip strength, and post-exercise lactate—while exerting only a modest effect on muscle structure. The two interventions produced largely independent effects, with cannabigerol’s synergy with training confined to power output. These findings position cannabigerol as a metabolic and neuromuscular modulator that complements exercise-induced adaptation rather than promoting hypertrophy. Given that sarcopenia and glucocorticoid-induced atrophy remain major clinical challenges, cannabigerol warrants further investigation as an adjunct to exercise; however, its mechanisms, generalizability to females and aged animals, and translation to age-related sarcopenia require dedicated study.

## Data Availability

All data supporting this study are available at the Open Science Framework (OSF): https://osf.io/df328/. The dataset is provided in tidy format under a Creative Commons Attribution 4.0 International (CC BY 4.0) license, enabling full reproducibility.

## Abbreviations

ANOVA: analysis of variance
AUC: area under the curve
CB1: cannabinoid receptor type 1
CB2: cannabinoid receptor type 2
CBD: cannabidiol
CBG: cannabigerol
CEUA: Comissão de Ética no Uso de Animais (Institutional Committee for Animal Care and Use)
CI: confidence interval
CK: creatine kinase
Cmax: peak concentration
CONCEA: Conselho Nacional de Controle de Experimentação Animal (Brazilian National Council for the Control of Animal Experimentation)
CSA: cross-sectional area
cyclo-CBG: cyclo-cannabigerol
DEXA: dexamethasone
H&E: hematoxylin and eosin
HSD: honestly significant difference
i.p.: intraperitoneal
OSF: Open Science Framework
PPAR: peroxisome proliferator-activated receptor
RT: resistance training
SED: sedentary
SEM: standard error of the mean
TRP: transient receptor potential
